# A case of malonyl coenzyme A decarboxylase deficiency with novel mutations and literature review

**DOI:** 10.3389/fped.2023.1133134

**Published:** 2023-04-17

**Authors:** Cong Zhao, Hua Peng, Nanchuan Jiang, Yalan Liu, Yan Chen, Jie Liu, Qing Guo, Zubo Wu, Lin Wang

**Affiliations:** ^1^ Department of Pediatrics, Union Hospital, Tongji Medical College, Huazhong University of Science and Technology, Wuhan, China; ^2^ Department of Radiology,Union Hospital, Tongji Medical College, Huazhong University of Science and Technology, Wuhan, China

**Keywords:** MCD, MLYCD, malonyl coenzyme A decarboxylase deficiency, developmental retardation, improvement of cardiomyopathy

## Abstract

**Introduction:**

Malonyl coenzyme A decarboxylase deficiency is caused by an abnormality in the MLYCD gene. The clinical manifestations of the disease involve multisystem and multiorgan.

**Methods:**

We collected and analyzed a patient's clinical characteristics, genetic chain of evidence and RNA-seq. We use the search term “Malonyl-CoA Decarboxylase Deficiency” on Pubmed to collect cases reported.

**Results:**

We report a 3-year-old girl who is presented with developmental retardation, myocardial damage and elevated C3DC. High-throughput sequencing identified heterozygous mutation (c.798G>A, p.Q266?) in the patient inherited from her father. The other heterozygous mutation (c.641+5G>C) was found in the patient inherited from her mother. RNA-seq showed that there were 254 differential genes in this child, among which 153 genes were up-regulated and 101 genes were down-regulated. Exon jumping events occurred in exons encoding PRMT2 on the positive chain of chromosome 21, which led to abnormal splicing of PRMT2. (P<0.05, FDR<0.05). The result of SNP showed that there were multiple mutation sites on chromosome 1, which may affect the downstream gene variation at the DNA level. The literature review identified 54 cases described since 1984.

**Discussion:**

It is the first report about the locus, adding a new item to the MLYCD mutation library. Developmental retardation and cardiomyopathy are the most common clinical manifestations, with commonly elevated malonate and malonyl carnitine levels in children.

## 1. Background

**Figure 1:**
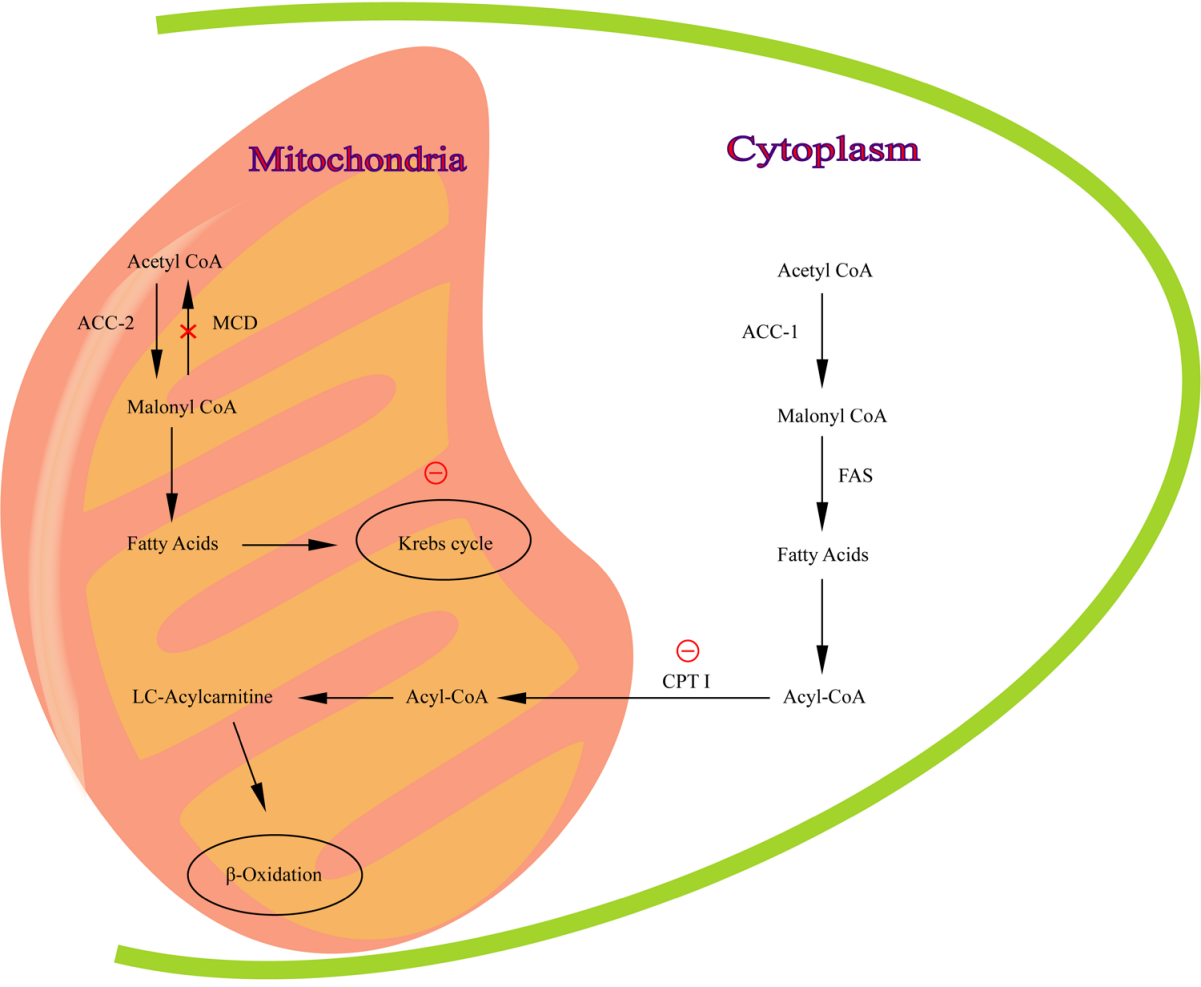
The pathway of malonyl-CoA regulation of fatty acid metabolism. MCD catalyzes the conversion of malonyl -CoA to acetyl-CoA in mitochondria. Red circle, inhibition of CPT I and Krebs cycle by malonyl-CoA.

The malonyl-CoA decarboxylase gene (*MLYCD*; MIM 606761) is located on chromosome 16 (16q23.3) (1), containing five exons and three possible promoters. The *MLYCD* gene encodes malonyl coenzyme A decarboxylase (MCD; EC 4.1.1. 9), which plays a crucial role in the regulation of lipid metabolism (2). MCD, presenting in mitochondria, peroxisomes and cytoplasm (3–5), is involved in the synthesis and oxidation of fatty acids to regulate lipid metabolism. MCD is most highly expressed in the myocardium, in addition to several organs such as skeletal muscle, brain, liver, pancreas, small intestine and kidney (3). It has been suggested that MCD may be important in the regulation of intracellular malonyl-CoA concentration (6).*β*-oxidation is inhibited when MCD is deficient (Figure 1).

When MCD is expressed abnormally, disease with multisystem and multiorgan damage (3, 7), occurs. This series of systemic symptoms caused by an abnormality in the *MLYCD* gene is called malonyl-CoA decarboxylase deficiency (MLYCDD; OMIM 248360). The manifestations are mainly about developmental retardation, epilepsy, hypotonia, hypertrophic cardiomyopathy, diarrhea, abdominal pain, constipation, vomiting, metabolic acidosis, hypoglycemia, ketosis, hyperlactatemia, etc., In some patients, brain imaging may reveal cerebral white matter lesions, giant cerebral gyrus, nodal ectopia, etc., as well as significantly low mitochondrial MCD activity in fibroblasts, and malonic aciduria (8, 9). Currently, specific treatment guidelines for MLYCDD are not available.

We report a 3-year-old child with dilated cardiomyopathy with “large motor backwardness and developmental retardation”. We found a mutation in the *MLYCD* gene according to the American College of Medical Genetics (ACMG) variant classification guidelines. This case reveals two novel loci mutations that lead to pathogenic alterations, updating and enriching the MLYCDD case pool. Further RNA seq analysis indicates there are 254 genes differentially expressed, among which 153 genes were up-regulated and 101 genes were down-regulated. Exon jumping event occurred on chromosome 21 in this child, which led to abnormal PRMT2 splicing. This patient is currently under follow-up and in a fair condition. We hope to obtain positive implications from the diagnosis, treatment, RNA-seq, for the early detection and new treatment of this disease.

## 2. Methods

### 2.1. Clinical characteristics

We obtain detailed information about the patient's birth characteristics, past medical history, personal history, family history, abnormal manifestations and examination results from the child's families.

### 2.2. Genetic chain of evidence

Screening of urinary organic acids by GC-MS ShimadzuQP-2010plus instrument. High precision clinical lineage testing is refined. Gene coding regions were amplified by a target region capture method and sequenced by a second-generation sequencing platform in this study. The genetic variants within the test range according to the ACMG variant classification guidelines and supplemental guideline.

### 2.3. RNA-seq analysis

We took the peripheral blood of this patient and the control group performed RNA-seq analysis. The control group involved in 3-year-old children without disease during physical examination. To determine differential gene expression, the read counts from each sequenced library were scaled normally by edgeR program package before differential gene expression analysis. A corrected *P*-value of 0.05 and an absolute fold change of 2 were set as the threshold for significantly different expression using the Benjamini & Hochberg method. There was a significant enrichment of differentially expressed genes in GO terms that had a corrected *P* value less than 0.05. A key mechanism to regulate gene expression and protein variables is alternative splicing. rMATS (3.2.5) software was used to analyze the AS event. GenBank ref seq nos: HG38, NM_012213.3.

### 2.4. Literature review

Using the search term “ Malonyl-CoA Decarboxylase Deficiency”, 32 case reports were retrieved on Pubmed. The articles related to combined malonic acid and methylmalonic aciduria were excluded. The total number of reported cases in the literature was 54.

## 3. Results

### 3.1. Basic information

The child was born at full term with no high-risk birth factors, no intrauterine growth abnormalities, no significant abnormalities in Apgar score or general condition. There were no history of close family members and genetic or infectious diseases. The child's growth and development were followed up regularly after birth.

### 3.2. Clinical characteristics

When the patient was 8 months old, she came to the hospital with fever. Unexpectedly, the cardiac ultrasound showed five questions: (1) spherical left ventricular dilatation with myocardial hypertrophy and abnormal echogenicity; (2) localized left ventricular noncompaction (LVNC); (3) left heart failure (EF 30%); (4) small ventricular septal defect (myocardial); (5) mild to moderate mitral and tricuspid valve insufficiency. The patient's growth and development were normal, so genetic examination was not arranged. After this pneumonia condition improved, she was given some pills for cardiac problem. The cardiac ultrasound was monitored periodically. The ultrasound results are shown in Table 1. In the following months, the patient hospitalized repeatedly for cardiomyopathy.

**Table 1:** Cardiac echocardiography of the child.

Age	LA	LV	RA	RV	FS	EF	LVEF(Simpson`s)
8 m	2.2	4.0	2.0	1.8	/	/	30%
9 m	1.5	3.9	1.6	1.4	/	/	36%
12 m	2.0	3.7	2.0	2.3	/	/	30%
16 m	1.9	3.2	1.8	1.7	/	/	32%
24 m	1.8	3.0	1.9	1.7	/	/	45%
33 m	1.8	2.9	2.3	2.2	37%	67%	/
38 m	1.7	2.9	2.1	1.8	36%	67%	/

Age: the age at which the examination was completed; LA, Left atrium; LV, Left ventricle; RA, Right atrium; RV, Right ventricle; FS, fractional shortening; EF, Ejection fraction; LVEF, left ventricular ejection fraction.

At one and a half years of age, the child was found to developmental retardation, mainly in talking and walking. But it had not yet affected the appetite. Meanwhile, the patient appeared uncontrolled twitch of lambs, which could stop on its own sometimes. We chose to continue to observe and assess her development regularly.

At two years of age, the patient still couldn't walk. What's more, she had a lower weight compared to children at the same age. Therefore, the patient was taken to the hospital for further inspection. The patient's blood tests indicated low blood glucose, elevated triglycerides and LDL cholesterol. A complete blood and urine organic acid metabolism test was arranged. Tandem mass speetrometry (MS/MS) showed palmitic acid-1: 152. 92 (>13. 8) and gas chromatography massspectrometry (GC/MS) showed C3DC/C4: 6.238uM (>2uM). The genetic examination was subsequently refined. The cranial MRI did not reveal any significant abnormalities. The diagnosis is on the basis of genetic results combined with clinical manifestations and auxiliary examinations.

### 3.3. Genetic chain of evidence

**Figure 2:**
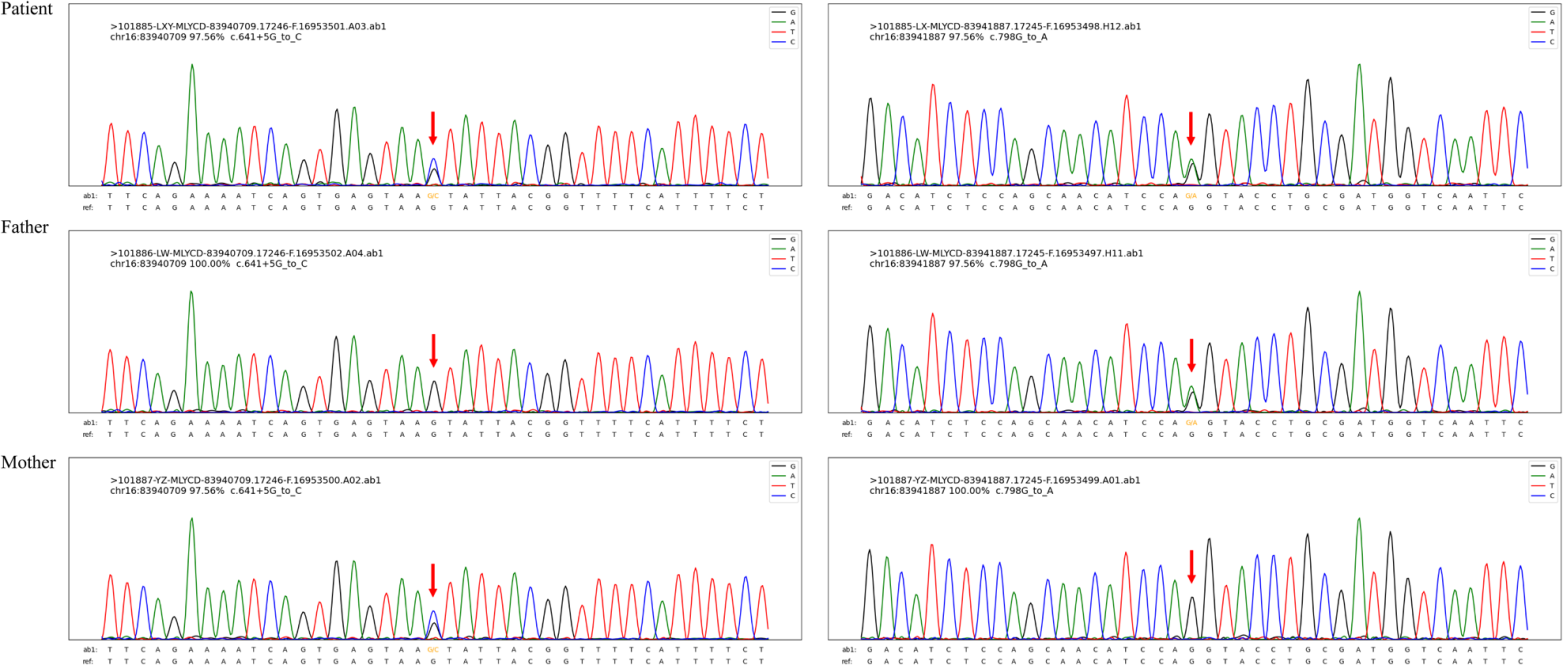
Dot variation correlation map of the MLYCD gene in the case family.

The *MLYCD* gene of the child showed c. 641 + 5 G > C, c. 798 G > A (p. Q266?) (HG19). The former derived from the maternal chromosome and the latter from the father's (Figure 2). The PP3-C1 and multiple computationally assisted algorithms were used to predict a higher likelihood of these two variants affecting splice function.

### 3.4. RNA-seq analysis

**Figure 3:**
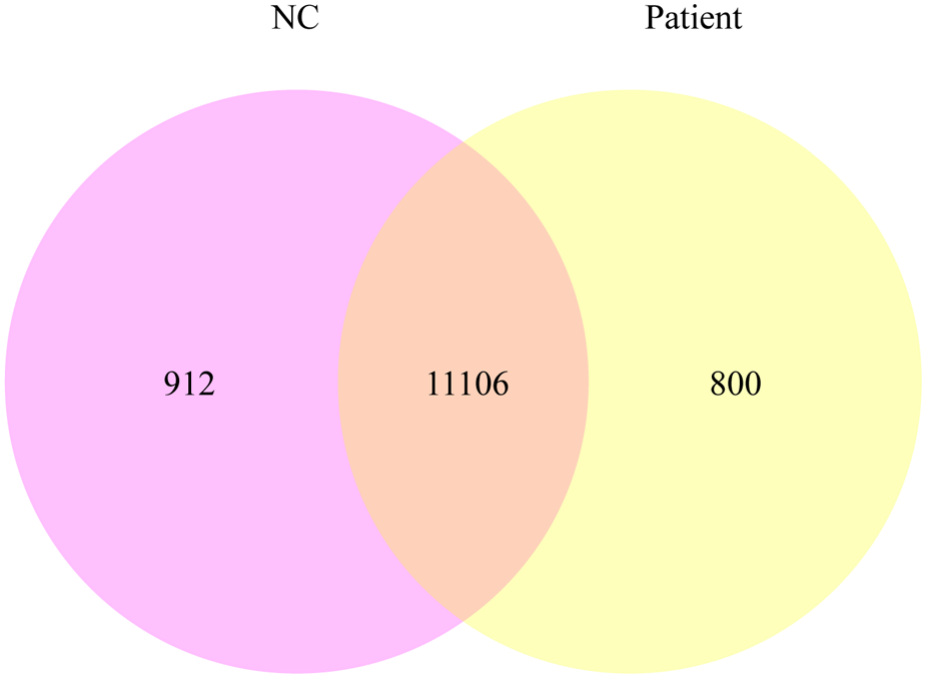
The co-expression Venn diagram shows the number of genes that are uniquely expressed in each group, and the overlapping regions show the number of genes that are co-expressed in two groups.

**Figure 4:**
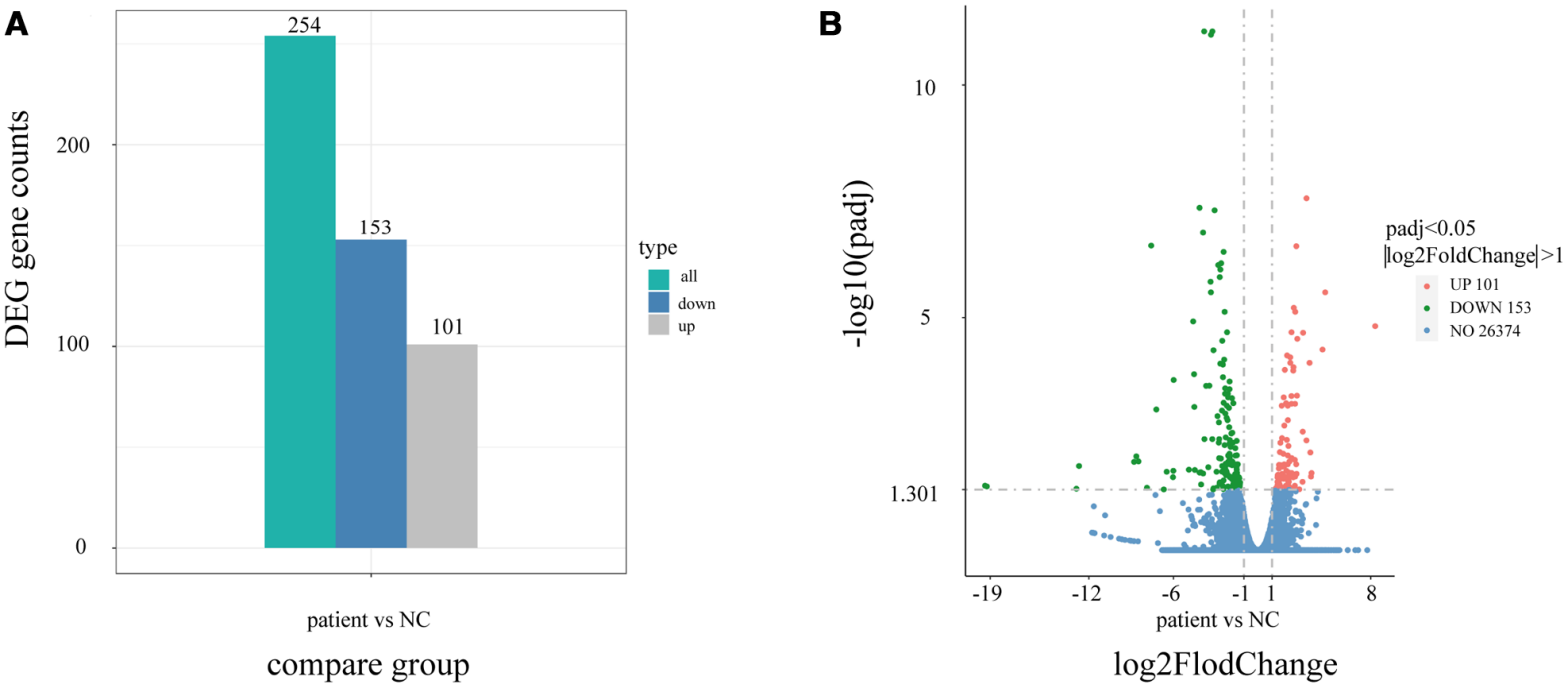
Differential gene statistical map. (**A**) Statistical histogram of the number of differential genes in different combinations. Blue and gray represent the up-regulated and down-regulated differential genes, respectively, and the numbers on the column indicate the number of differential genes. (**B**) Differential gene volcano map. In the picture, the Abscissa is present log2FoldChange, the ordinate is present -log10padj or -log10pvalue, and the blue dotted line represents the threshold line for differential gene screening criteria.

Using quantitative gene expression analysis, we found that 11,106 genes were expressed in the normal (control group) and this patient (Figure 3). Quantification of gene expression is followed by statistical analysis to determine genes with significantly different expression levels across states. The differentially expressed genes (DEGs) of the two groups were compared, and there were 254 differential genes in this child, among which 153 genes were up-regulated and 101 genes were down-regulated (Figure 4).

**Figure 5:**
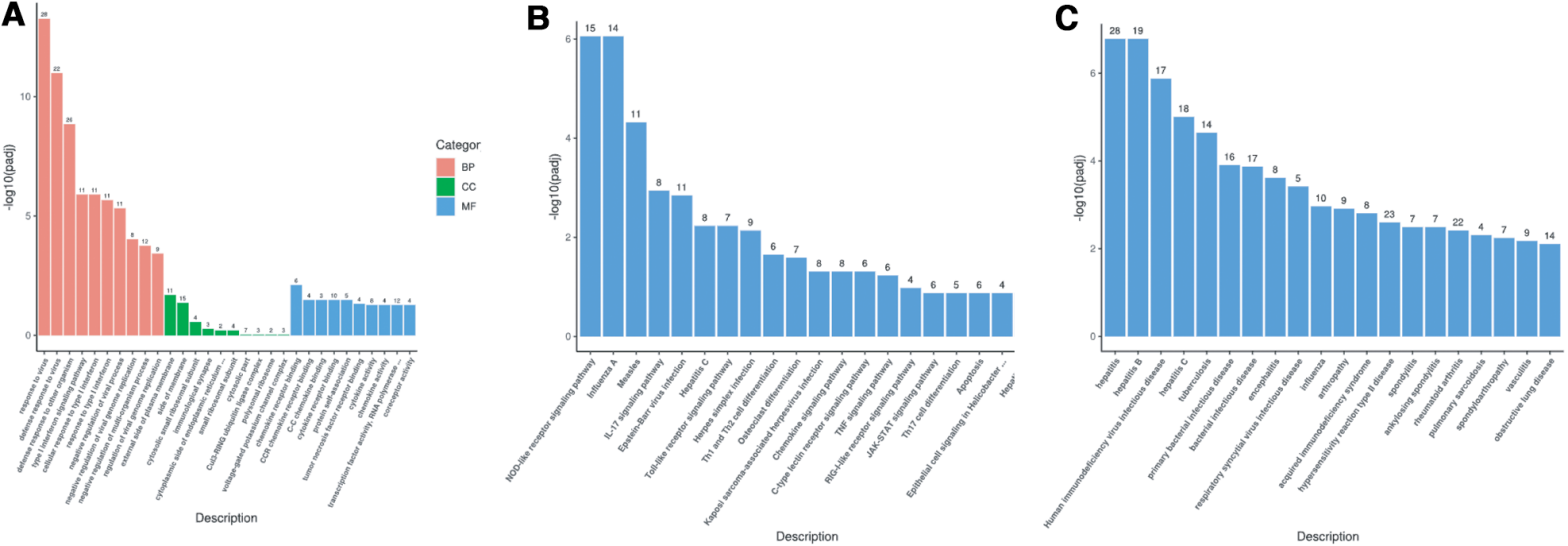
(**A**) GO enrichment analysis bar chart; (**B**) KEGG enrichment analysis bar chart; (**C**) DO enrichment analysis bar chart.

Gene Ontology (GO) enrichment analysis of the above differential genes showed that the child had more obvious effects on biological processes such as response to virus, defense response to virus and the defense response to other organisms, were more obvious (Figure 5A). The results of KEGG pathway enrichment analysis show that the enrichment degree of NOD-like receptor signaling pathway and Influenza A pathways is significant, which is worthy of further study (Figure 5B). Through Disease Ontology analysis, we found that the child had a higher risk of hepatitis and immunodeficiency disease. (*P* < 0.001) (Figure 5C).

In addition to expression analysis, we also use RNA-seq data to analyze alternative splicing, SNP/indel and other variant sites according to the alignment of the child's gene with the reference genome. Previous studies have suggested that mutations in introns may lead to incorrect splicing (2). In the patient we reported, 176 exons jumping events occurred in exons encoding protein arginine methyltransferase 2 (PRMT2) on the positive chain of chromosome 21, which led to abnormal splicing of PRMT2. (*P* < 0.05, FDR < 0.05) The sequence shows following: TTTGAGTTCATGATCGAGTCCATC CTGTATGCCCGGGATGCCTGGCTGAAGGAGGACGGGGTCATTTGGCCCACCATGGCTGCGTTGCACCTTGTGCCCTGCAGTGCTGATAAGGATTATCGTAGCAAGGTGCTCTTCTGGGACAACGCGTACGAGTTCAACCTCAGCGCTCTGAA. The results of SNP showed that there were multiple mutation sites on chromosome 1, which may affect the downstream gene variation at the DNA level. (Supplementary Table). Combined with the clinical presentation of the child and its family lineage analysis, the clinical correlation was strong, and the current clinical presentation of the child was considered to be caused by the *MLYCD* gene variant. It was classified as pathogenic according to the recommendations of the American College of Medical Genetics (ACMG) (10).

### 3.5. Patient treatment

The treatment for this patient was divided into three parts: (1) Cardiac treatment and dietary controlling. Digoxin (5 ug/kg/d), milrinone (0.5 ug/kg/min), diuretic such as furosemide or spironolactone (2 g/kg/d), angiotensin-converting enzyme inhibitors and prednisone were used to improve cardiac function and reduce cardiac load. Some adjuvant drugs such as coenzyme Q10 and vitamin B were used to treat the patient as well. Throughout the whole treatment, immunoglobulin was supported at small dose many times; (2) Convulsion controlling. DeBakin was used to control the epileptic seizure; (3) Diet adjustment. Carnitine supplementation (levocarnitine, 50 mg/kg/day) was introduced. After diagnosis, low-fat, high-carbohydrate, medium-chain fatty acid and L-carnitine were given.

After the treatment above, the patient's heart function recovered gradually. Convulsions decreased even disappeared as well. Her blood sugar, triglycerides and LDL can be maintained in the normal range in the last two years of physical examination. and now it is possible to walk. Particularly, the patient grows gradually and she can walk as normal now.

### 3.6. Literature review

MLYCDD was first proposed by Brown et al. in 1984, and 54 cases of MLYCDD were reported worldwide. The most common symptoms in these patients were developmental retardation (77.6%, *n* = 38 out of 49), dystonia (82.6%, *n* = 19 out of 23) and talk delay (85.0%, *n* = 17 out of 20). Cardiomyopathy are serious complication. Among patients reported, there were 40 in total with urinary malonic acid. Hypoglycemia (*n* = 54.8%,17 out of 31) can be considered acute manifestations. There are only 4 patients with left ventricular noncompaction, making the patient we reported here the fifth reported MLCYD patient with this finding (Table 2). The usual time to discover the disease is neonatal period. A neonatal presentation with feeding difficulties, failure to thrive were common initial clinical manifestation.

**Table 2:** Manifestation and auxiliary examination of reported children with MLYCDD.

Symptom	Number	Rate (%)	Symptom exist
Developmental retardation	38	38/49 (77.6)	Yes
Growth retardation	25	25/40 (62.5)	Yes
Epileptic seizure	17	17/39 (43.6)	Yes
Close parents	18	18/32 (56.3)	No
Dystonia	19	19/23 (82.6)	Yes
Talk delay	17	17/20 (85.0)	Yes
Feeding difficulty	10	10/13 (76.9)	No
Microcephaly	9	9/12 (75.0)	No
Renal dysplasia	2	2/10 (20.0)	No
Digestive system symptoms	9	9/9 (100.0)	No
Lethargy	4	4/6 (66.7)	No
Brain MRI	13	13/22 (59.1)	No
Urinary malonic acid	40	40/40 (100.0)	ND
Urinary methyl malonic acid	28	28/32 (87.5)	ND
Hypoglycemia	17	17/31 (54.8)	Yes
C3DC	18	18/18 (100.0)	Yes

Previously reported cases (*n* = 54); Number: the number of patients who had this symptom; Symptom exist: Whether the patient we reported had the symptoms; ND, not done.

There are elevated levels of malonic acid in urine as well as malonyl carnitine (C3DC) in plasma or whole blood that can be used to diagnose malonic aciduria. Among patients reported, there were 18 (100%, *n* = 18) patients with urinary malonic acid. There are forty-seven unique variants reported in the literature (Table 3). The variant reported here has never been described. Diagnosis of malonic aciduria can get the evidence from elevations of malonyl-carnitine and malonic acid in metabolic investigations. Combining genetic changes and a series of systemic manifestations, MLYCDD can be diagnosed affirmatively. The treatment can be classified as two aspects: (1) Etiological treatment. Patients with MLYCDD have been treated with a fat restricted diet. The specific diet, which is lower in long-chain fatty acids and richer in carbohydrates and medium-chain triglycerides than usual (11–14). Carnitine supplementation was necessary; (2) Symptomatic treatment. The dilated cardiomyopathy was treated with furosemide, captopril and angiotensin converting enzyme (ACE) (7, 12).

**Table 3:** Conditions of pathogenic genes in children affected with MLYCDD.

References	MLYCD mutations	References	MLYCD mutations
Sarah et al.	c.346C > T	Salomons et al.	c.560C > G; Deletion exons 1 to 5
	c.346C > T		c.796C > T
Kasapkara et al.	c.175A > T		c.796C > T
Lee et al.	c.1A > G; Deletion exons 1 to 3		c.481del
Cristel et al.	Deletion exon 1 and 5′UTR		5′UTR deletion
	c.640_641 + 2del		c.1319G > T; c.1367A > C
	Deletion exon 5		c.949-14A > G
	c.365T > C; c.1152G > T		c.899G > T; c.1430C > T
	Deletion exon 5		c.799-1983_949-1293del
	c.641 + 4_641 + 7del	Malvagia et al.	c.772_775del
	c.22_34dup; c.799-3C > G	Salomons et al.	c.560C > G; Deletion exons 1 to 5
	c.929G > C	Wightman et al.	c.59dup; c.634dup
	Deletion exon 5		c.59dup; c.634dup
Ersoy et al.	c.13_14insG		c.1151_1159del
Liu et al.	Deletion exons 1 to 3; c.911G > A		c.206C > T
Polinati et al.	c.2T > A; c.454C > A		c.482T > C
Baertling et al.	Deletion exons 1 to 5	Gao et al.	c.638_641del
	Deletion exons 1 to 5	Yano et al.	c.84_108del
Celato et al.	c.672G > A; c.869C > T	Krawinkel et al.	c.393C > G
Xue et al.	c.920T > G; Deletion exon 1	Matalon et al.	c.119T > C
Prada et al.	c.393_400del		c.949-14A > C
Footitt et al.	c.197del		c.560C > G
Brown et al.	c.8G > A	Haan et al.	c.475del

## 4. Discussion

The first case of MLYCDD was reported in 1983. Fifty-four cases have been reported in the literature (12) up to now. Just as the case described in this paper, the gene variance belongs to a new causative locus. The patient had cardiac abnormalities as the first manifestation. Psychiatric and neurological symptoms appeared later. At present, general condition of the patient is relatively stable, and the long-term follow-up is planned. We can see that genetic testing is extremely important for disease with atypical symptom. With a small number of reported cases and unidentical genetic variants sites, genetic testing among children with a high suspicion of MLYCDD may be meaningful.

Due to the abnormal *MLYCD* gene in this patient, MCD can't be synthesized properly. As a result, malonyl-CoA is not converted properly to acetyl-CoA. Malonyl-CoA can be used as a raw material to form new palmitoyl salts using fatty acid synthase in some adipo-genic tissues such as liver, adipose tissue and lactating glands. If there are something wrong with MCD biosynthesis, palmitic acid levels will be elevated as the patient we reported. Therefore, MS/MS and GC/MS are recommended when hereditary diseases are suspected.

As we all know, energy requirements of the organs are usually met by oxidation of glucose and fatty acids. It has been shown that mitochondrial oxidation of fatty acid is impaired in MLYCDD patients’ cultured fibroblasts (6). CPT I inhibition is responsible for reduced metabolic flow (6, 15). Malonyl-CoA is a potent endogenous inhibitor of CPTI., When the concentration of malonyl-CoA is low, the muscle isomer of CPT I is inhibited in the myocardium (6), oxidation of myocardial fatty acid is thus inhibited. As a result, myocardium will be impaired by hypoxia. The patient reported here with cardiac damage at an earlier age was consistent with abnormal metabolic manifestations of the disease. Therefore, patients with MLYCDD should be alert to cardiac manifestations and required cardiac ultrasound monitoring. From the statistics of reported cases, we found that 76.9% (*n* = 10 out of 13) patients had feeding difficulties. It suggests that when neonatal feeding difficulties are found during clinical work, hereditary diseases need to be taken into account as well. At the same time, cardiac ultrasound is recommended. More importantly, our patient suffered cardiomyopathy first and other problems were revealed after then. In the cases reported, 62.5% (*n* = 25 out of 40) patients have heart involvement such as dilated cardiomyopathy, LVNC and so on. Despite the fact that the first symptoms haven't always been the same in the reported cases, cardiac damage deserves high priority and regular monitoring.

When CPT I deficiency in liver occurred, it could cause liver failure and hypoglycemic disorders (16). Our patient had hypoglycemia during the course, but no liver failure had been reported yet. This is one of the priorities that require long-term monitoring. Since MLYCDD lacks specific signs and is often associated with developmental retardation, clinical diagnosis is difficult. The common diagnostic methods of MLYCDD are MS/MS, GC/MS and genetic testing. It is equally important to screen subsequently.

It's the first time to carry on RNA-seq aimed to MLYCDD patient. RNA-seq is a developed method to transcriptome profiling that uses deep-sequencing technologies (17). By analyzing genomic data, more modifier genes can be gained from a particular genetic background. Unlike genetic testing, it enables the identification and quantification of RNA molecules from a biological sample. Alternative Splicing (AS) and DEGs at the mRNA level can be analyzed by the tool. As RNA-Seq is quantitative, it can be used to determine RNA expression levels more accurately. At the same time, it will allow robust comparison between diseased and normal tissues, as well as the subdivision of disease states.

AS showed that the patient had abnormal protein arginine methyltransferase 2 (PRMT2) shearing (*P* < 0. 05). Studies have shown that PRMT2 has the ability to promote apoptosis, and to be involved in energy homeostasis (18). PRMT2 is associated with disorders of energy metabolism, obesity resistance and leptin sensitivity (19, 20). PRMT2 represents a glucose-sensitive factor controlling ABCA1-dependent cholesterol efflux (21), while it has potential to explain atherosclerosis in diabetic patients (22). No study has revealed the relationship between PRMT2 and MLYCD. However, in the case reported here, PRMT2 shearing was abnormal and statistically significant, which suggested an association between PRMT2 and MLYCD. The author speculates that as a result of an error in gene expression, PRMT2 is not expressed correctly in the patient, leading to a variety of symptoms. But it still needs to be further confirmed, which provides a new idea for later studies.

It has been shown that dietary interventions have positive implications for the disease controlling in children. Restricted supplementation of long-chain triglycerides and medium-chain triglycerides (30% LCT, 70% MCT) in children, added with ACEI has been shown to be beneficial in improving cardiac function in children, based on imaging evidence (23). In the case reported here, we gave the child ACEI oral medication. But it is unclear which of the two, dietary control or angiotensin-converting enzyme inhibition, is more effective in controlling the progression of cardiomyopathy. Further research can be conducted based on these findings. Studies have shown that appropriate carnitine supplementation (5 milligram per kilogram, in a day) can make symptoms such as vomiting, acidosis, and liver damage gradually better (24). Clinical conditions can be improved when the diet is changed before the onset of significant disease symptoms.

As a serious genetic disorder, MLYCDD is currently not covered in newborn metabolic monitoring program in recent years. Studies have shown that monitoring and early-intervention in the neonatal period are necessary (25). Authors conclude that enhanced genetic counseling and prenatal diagnosis are necessary, although substantial clinical evidence is lacking for newborn screening for MCD deficiency. However, recent results suggest that MS/MS and GC/MS screening would be valuable for the disease early-intervention for newborn (25), In the case, early detection and intervention are particularly important. Early but effective screening can help to obtain more direct clinical evidence of early and effective intervention, which has positive implications for the prognosis of the patients. Earlier intervention may be advantageous in avoidance of life-threatening clinical findings, including cardiomyopathy (25).

## Data Availability

The datasets for this article are not publicly available due to concerns regarding participant/patient anonymity. Requests to access the datasets should be directed to the corresponding author.
