# Corrigendum: A case of malonyl coenzyme a decarboxylase deficiency with novel mutations and literature review

**DOI:** 10.3389/fped.2023.1339656

**Published:** 2023-11-27

**Authors:** Cong Zhao, Hua Peng, Nanchuan Jiang, Yalan Liu, Yan Chen, Jie Liu, Qing Guo, Zubo Wu, Lin Wang

**Affiliations:** ^1^ Department of Pediatrics, Union Hospital, Tongji Medical College, Huazhong University of Science and Technology, Wuhan, China; ^2^ Department of Radiology, Union Hospital, Tongji Medical College, Huazhong University of Science and Technology, Wuhan, China

**Keywords:** MCD, MLYCD, malonyl coenzyme a decarboxylase deficiency, developmental retardation, improvement of cardiomyopathy

A Corrigendum on A case of malonyl coenzyme a decarboxylase deficiency with novel mutations and literature review By Zhao C, Peng H, Jiang N, Liu Y, Chen Y, Liu J, Guo Q, Wu Z and Wang L (2023) Front. Pediatr. 11:1133134. doi: 10.3389/fped.2023.1133134

In the published article, there was an error in affiliation 1, “Department of Pediatrics, Wuhan Union Hospital, Tongji Medical College, Huazhong University of Science and Technology, Wuhan, China” has been changed to “Department of Pediatrics, Union Hospital, Tongji Medical College, Huazhong University of Science and Technology, Wuhan, China”.

The authors apologize for this error and state that this does not change the scientific conclusions of the article in any way. The original article has been updated.

