# Supplementary material for: A case of malonyl coenzyme A decarboxylase deficiency with novel mutations and literature review
Source: Front Pediatr. 2023 Apr 17;11:1133134. doi: 10.3389/fped.2023.1133134 (PMC10152364; doi:10.3389/fped.2023.1133134)
Supplement: Supplementary file 1 [file Table1.docx]

| CHROM | REF | ALT | QUAL | Gene Name | Biotype | HGVS C | EFFECT | IMPACT |
| --- | --- | --- | --- | --- | --- | --- | --- | --- |
| 1 | T | A | 335.6 | WASH7P | unprocessed pseudogene | n.*791A>T | downstream gene variant | MODIFIER |
| 1 | T | A | 335.6 | MIR6859-1 | miRNA | n.*3756A>T | downstream gene variant | MODIFIER |
| 1 | T | A | 335.6 | DDX11L1 | processed transcript | n.861T>A | non coding transcript exon variant | MODIFIER |
| 1 | T | A | 335.6 | DDX11L1 | transcribed unprocessed pseudogene | n.575T>A | non coding transcript exon variant | MODIFIER |
| 1 | A | C | 621.6 | WASH7P | unprocessed pseudogene | n.*499T>G | downstream gene variant | MODIFIER |

Single Nucleotide Polymorphisms of the patient

CHROM: The chromosome number where the mutation site is located; REF: The base of a mutation site in the reference genome; ALT: The actual base of the mutation site in the sample; QUAL: The quality of the variation site, the value of the Phred format, the higher the value, the higher the reliability of the variant site genotype; Gene Name: The name of the gene in which the mutation site is located; Biotype: The type of transcript where the mutation site is located; HGVS C: HGVS annotations of variation sites at DNA level; EFFECT: The effect of the mutation site; IMPACT: The degree of influence caused by the mutation site
